# 4-Nitro­anilinium triiodide monohydrate

**DOI:** 10.1107/S1600536810014674

**Published:** 2010-04-28

**Authors:** David G. Billing, Robert S. Black, Wonga M. Hexana

**Affiliations:** a Molecular Sciences Institute, School of Chemistry, University of the Witwatersrand, Private bag, PO Wits 2050, South Africa

## Abstract

In the title compound, C_6_H_7_N_2_O_2_^+^·I_3_^−^·H_2_O, the triiodide anions form two-dimensional sheets along the *a* and *c* axes. These sheets are separated by the 4-nitro­anilinium cations and water mol­ecules, which form part of an extended hydrogen-bonded chain with the triiodide along the *c* axis, represented by the graph set *C*_3_^3^(14). The second important hydrogen-bonding inter­action is between the nitro group, the water mol­ecule and the anilinium group, which forms an *R*_2_^2^(6) ring and may be the reason for the deviation of the torsion angle between the benzene ring and the nitro group from 180 to 163.2 (4)°. These two strong hydrogen-bonding inter­actions also cause the benzene rings to pack off-centre from one another, with an edge-on-edge π–π stacking distance of 3.634 (6) Å and a centroid–centroid separation of 4.843 (2) Å.

## Related literature

For structures of 4-nitro­anilinine-monohalide salts, see: Lemmerer & Billing (2006 ▶) (bromine) and Ploug-Sørensen & Andersen (1982 ▶) (chlorine). For other amine-based triiodide salts, see: Tebbe & Loukili (1998 ▶). For a triiodide salt containing a tetra­phenyl­phospho­nium cation, see: Parvez *et al.* (1996 ▶). For structure-properties relationships in trihalides, see: Shibaeva & Yagubskii (2004 ▶). For graph-set analysis, see: Etter *et al.* (1990 ▶). 
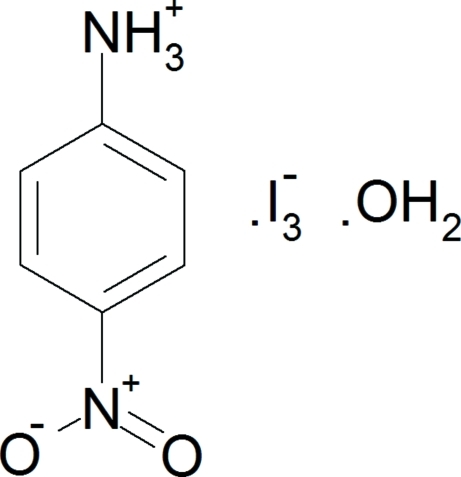


## Experimental

### 

#### Crystal data

C_6_H_7_N_2_O_2_^+^·I_3_^−^·H_2_O
*M* *_r_* = 537.85
Monoclinic, 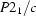

*a* = 4.8429 (9) Å
*b* = 14.701 (3) Å
*c* = 18.346 (3) Å
β = 91.916 (3)°
*V* = 1305.4 (4) Å^3^
*Z* = 4
Mo *K*α radiation
μ = 7.17 mm^−1^
*T* = 298 K
0.54 × 0.31 × 0.11 mm

#### Data collection

Bruker SMART 1K CCD area-detector diffractometer
Absorption correction: integration (*XPREP*; Bruker, 1999 ▶) *T*_min_ = 0.113, *T*_max_ = 0.506
8741 measured reflections
3150 independent reflections
2461 reflections with *I* > 2σ(*I*)
*R*_int_ = 0.068

#### Refinement

*R*[*F* ^2^ > 2σ(*F* ^2^)] = 0.034
*wR*(*F* ^2^) = 0.081
*S* = 1.05
3150 reflections
137 parameters
H atoms treated by a mixture of independent and constrained refinement
Δρ_max_ = 0.71 e Å^−3^
Δρ_min_ = −1.42 e Å^−3^

### 

Data collection: *SMART-NT* (Bruker, 1998 ▶); cell refinement: *SMART-NT*; data reduction: *SAINT-Plus* (Bruker, 1999 ▶); program(s) used to solve structure: *XS* in *SHELXTL* (Sheldrick, 2008 ▶); program(s) used to refine structure: *SHELXL97* (Sheldrick, 2008 ▶); molecular graphics: *ORTEP-3 for Windows* (Farrugia, 1997 ▶) and *DIAMOND* (Brandenburg, 1999 ▶); software used to prepare material for publication: *WinGX* (Farrugia, 1999 ▶) and *PLATON* (Spek, 2009 ▶).

## Supplementary Material

Crystal structure: contains datablocks global, I. DOI: 10.1107/S1600536810014674/zs2032sup1.cif

Structure factors: contains datablocks I. DOI: 10.1107/S1600536810014674/zs2032Isup2.hkl

Additional supplementary materials:  crystallographic information; 3D view; checkCIF report

## Figures and Tables

**Table 1:** Hydrogen-bond geometry (Å, °)

*D*—H⋯*A*	*D*—H	H⋯*A*	*D*⋯*A*	*D*—H⋯*A*
N2—H2*A*⋯O3^i^	0.89	1.94	2.824 (5)	173
N2—H2*B*⋯I3^ii^	0.89	3.01	3.731 (4)	139
N2—H2*C*⋯O1^iii^	0.89	2.52	2.922 (5)	108
N2—H2*C*⋯O3^iii^	0.89	2.02	2.860 (5)	157
O3—H3*A*⋯O2	0.88 (2)	1.98 (3)	2.818 (5)	158 (6)
O3—H3*B*⋯I1^iv^	0.89 (5)	2.88 (5)	3.722 (3)	157 (4)

Symmetry codes: (i) 
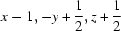
; (ii) 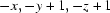; (iii) 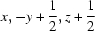; (iv) 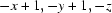.

**Fig. 1.**
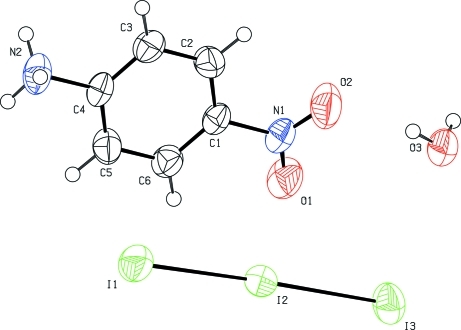
View of (I) (50% probability displacement ellipsoids)

**Fig. 2.**
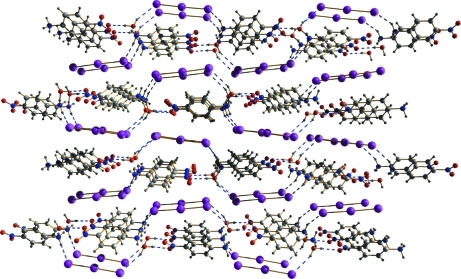
A view along the a axis of an extended unit cell showing the alignment of the triiodide moieties and C33(14) H-bonding interaction.

## supplementary crystallographic information

### Comment

Previously 4-nitroanilinine was crystallized with bromine (Lemmerer & Billing, 2006) and chlorine (Ploug-Sørensen *et al.*, 1982) to produce the respective monohalide salts. In an attempt to synthesize a monoiodide salt with 4-nitroaniline, the black crystals of 4-nitroanilinium triiodide monohydrate, C_6_H_7_N_2_O_2_^+^ I_3_^-^ . H_2_O (I) formed in preference, and the structure is reported here. Polyiodide salts are commonly found, but the triiodides less so. Tebbe & Loukili (1998) have successfully synthesized two tertiary ammonium triiodide salts, while Parvez *et al.* (1996) synthesized a tetraphenylphosphonium triiodide salt. This is important to note since the title compound has a primary amine as the cation, while in the other three reported cases, bulky counter cations are involved. There are no other structural similarities with (I) with the exception of the the I1—I2—I3 bond angle [178.209 (14)°], which compares with those of the tertiary ammonium triiodides (180, 177.09°) and the bulkier tetraphenylphosphonium triiodide (175.27°).

In the structure of (I) (Figs. 1, 2), the triiodide anions essentially form two-dimensional sheets along the *a* and *c* axes. Looking at the interactions along the *a* axis, the layers of triiodide anions pack parallel to each other with a separation of 4.843 (1) Å. The two intermolecular head-to-tail I1···I1 and the two I3···I3 interactions along the *c* axis have a separation of 4.574 (1) and 3.772 (1) Å and 4.1079 (7) and 5.2776 (8) Å respectively, completing the interactions which form the two-dimensional sheets. These sheets are separated by the 4-nitroanilium and water moieties which form part of an extended hydrogen-bonded chain with the triiodide along the *c* axis of the unit cell, represented by the graph set C^3^_3_(14) (Etter *et al.*, 1990). The graph set notation includes H···I hydrogen bonds with the water and the nitro oxygen (O2) *i.e.* (O3—H···O2), as seen in Fig 2.

Besides the strong C^3^_3_(14) hydrogen-bonding network, another important hydrogen-bonding association is between the nitro group, the water and the ammonium group, forming an R^2^_2_(6) ring (Table 1). This ring appears to be an important interaction which gives a deviation of the torsion angle C6—C1—N1—O2 between the benzene ring and the nitro group from 180° to 163.2 (4)°. The two strong hydrogen-bonding interactions result in the benzene rings packing off-centre from one another with an edge-on-edge π-π stacking distance of 3.634 (6) Å and a centroid-to-centroid separation of 4.843 (2) Å. The many short intermolecular distances between the triiodide anions and the benzene rings may be important in the optical properties of (I), regarding charge-transfer interactions and conductivity, as found in this type of compound (Shibaeva & Yagubskii, 2004).

### Experimental

For the preparation of (I) 0.632 g of 4-nitroaniline was dissolved in 4 ml of 55% aqueous HI. The solution was heated to dissolve the precipitate and then left to stand at room temperature. Crystals suitable for single crystal X-ray diffraction were grown by slow evaporation of the solvent over a period of one month.

### Refinement

The H atoms on nitroaniline were refined using a riding-model, with C—H = 0.93 Å, N—H = 0.89 Å and with *U*_ĩso_(H) = 1.2*U*_eq_(C) or 1.5*U*_eq_(N). The H atoms on the water were placed from the difference Fourier map with O—H = 0.90 (2) Å and constrained using the *DFIX* constraint (Sheldrick, 2008). The highest residual electron density peak (0.708eÅ^-3^) was 0.865 Å from I2.

### Crystal data

**Table:**

C_6_H_7_N_2_O_2_^+^·I_3_^−^·H_2_O	*F*(000) = 968
*M**_r_* = 537.85	*D*_x_ = 2.737 Mg m^−^^3^
Monoclinic, *P*2_1_/*c*	Mo *K*α radiation, λ = 0.71073 Å
Hall symbol: -P 2ybc	Cell parameters from 9074 reflections
*a* = 4.8429 (9) Å	θ = 2.6–28.3°
*b* = 14.701 (3) Å	µ = 7.17 mm^−^^1^
*c* = 18.346 (3) Å	*T* = 298 K
β = 91.916 (3)°	Plate, black
*V* = 1305.4 (4) Å^3^	0.54 × 0.31 × 0.11 mm
*Z* = 4	

### Data collection

**Table:**

Bruker SMART 1K CCD area-detector diffractometer	2461 reflections with *I* > 2σ(*I*)
φ and ω scans	*R*_int_ = 0.068
Absorption correction: integration (*XPREP*; Bruker, 1999)	θ_max_ = 28°, θ_min_ = 1.8°
*T*_min_ = 0.113, *T*_max_ = 0.506	*h* = −6→4
8741 measured reflections	*k* = −19→18
3150 independent reflections	*l* = −24→24

### Refinement

**Table:**

Refinement on *F*^2^	H atoms treated by a mixture of independent and constrained refinement
Least-squares matrix: full	*w* = 1/[σ^2^(*F*_o_^2^) + (0.0374*P*)^2^ + 0.3561*P*] where *P* = (*F*_o_^2^ + 2*F*_c_^2^)/3
*R*[*F*^2^ > 2σ(*F*^2^)] = 0.034	(Δ/σ)_max_ = 0.001
*wR*(*F*^2^) = 0.081	Δρ_max_ = 0.71 e Å^−^^3^
*S* = 1.05	Δρ_min_ = −1.42 e Å^−^^3^
3150 reflections	Extinction correction: *SHELXL97* (Sheldrick, 2008), Fc^*^=kFc[1+0.001xFc^2^λ^3^/sin(2θ)]^-1/4^
137 parameters	Extinction coefficient: 0.0166 (6)
0 restraints	

### Special details

**Table:**

Experimental. Numerical integration absorption corrections based on indexed crystal faces were applied using the XPREP routine (Bruker, 1999*a*)
Geometry. All esds (except the esd in the dihedral angle between two l.s. planes) are estimated using the full covariance matrix. The cell esds are taken into account individually in the estimation of esds in distances, angles and torsion angles; correlations between esds in cell parameters are only used when they are defined by crystal symmetry. An approximate (isotropic) treatment of cell esds is used for estimating esds involving l.s. planes.

### Fractional atomic coordinates and isotropic or equivalent isotropic displacement parameters (Å^2^)

**Table:**

	*x*	*y*	*z*	*U*_iso_*/*U*_eq_	
C1	0.4993 (8)	0.2871 (3)	0.2902 (2)	0.0404 (8)	
C2	0.6434 (9)	0.3443 (3)	0.3357 (2)	0.0491 (10)	
H2	0.7771	0.383	0.3179	0.059*	
C3	0.5861 (9)	0.3433 (3)	0.4094 (2)	0.0482 (10)	
H3	0.6779	0.3822	0.442	0.058*	
C4	0.3913 (8)	0.2838 (3)	0.4328 (2)	0.0410 (9)	
C5	0.2464 (9)	0.2267 (3)	0.3873 (2)	0.0502 (10)	
H5	0.1157	0.187	0.4053	0.06*	
C6	0.2991 (9)	0.2293 (3)	0.3129 (2)	0.0481 (10)	
H6	0.2006	0.1928	0.2798	0.058*	
N1	0.5625 (8)	0.2874 (3)	0.21247 (18)	0.0494 (9)	
N2	0.3444 (8)	0.2797 (3)	0.51204 (17)	0.0574 (10)	
H2A	0.1948	0.2464	0.5199	0.086*	
H2B	0.3198	0.3357	0.529	0.086*	
H2C	0.4903	0.2545	0.5349	0.086*	
O1	0.3995 (8)	0.2526 (3)	0.16900 (17)	0.0692 (10)	
O2	0.7804 (8)	0.3222 (3)	0.19569 (19)	0.0774 (11)	
O3	0.8528 (8)	0.3133 (2)	0.04409 (18)	0.0583 (8)	
H3A	0.878 (14)	0.316 (5)	0.0917 (12)	0.11 (3)*	
H3B	0.800 (13)	0.366 (3)	0.024 (3)	0.11 (2)*	
I1	0.33289 (8)	0.50637 (2)	0.091253 (18)	0.06519 (14)	
I2	0.22677 (6)	0.531920 (18)	0.244438 (16)	0.04997 (12)	
I3	0.12058 (7)	0.55190 (2)	0.402108 (17)	0.06257 (14)	

### Atomic displacement parameters (Å^2^)

**Table:**

	*U*^11^	*U*^22^	*U*^33^	*U*^12^	*U*^13^	*U*^23^
C1	0.047 (2)	0.045 (2)	0.0290 (18)	0.0061 (17)	0.0020 (16)	−0.0012 (16)
C2	0.055 (3)	0.051 (2)	0.041 (2)	−0.0106 (19)	0.003 (2)	0.0007 (18)
C3	0.057 (3)	0.055 (2)	0.0320 (19)	−0.003 (2)	−0.0039 (18)	−0.0060 (18)
C4	0.043 (2)	0.051 (2)	0.0292 (18)	0.0095 (17)	0.0039 (16)	0.0001 (16)
C5	0.049 (3)	0.062 (3)	0.041 (2)	−0.005 (2)	0.0075 (19)	0.002 (2)
C6	0.049 (2)	0.054 (2)	0.041 (2)	−0.0027 (19)	0.0015 (19)	−0.0070 (19)
N1	0.057 (2)	0.057 (2)	0.0341 (18)	0.0053 (18)	0.0045 (17)	−0.0046 (16)
N2	0.056 (2)	0.083 (3)	0.0341 (18)	−0.002 (2)	0.0071 (16)	−0.0006 (19)
O1	0.078 (2)	0.090 (3)	0.0390 (17)	−0.009 (2)	−0.0025 (16)	−0.0066 (17)
O2	0.072 (2)	0.115 (3)	0.0463 (19)	−0.020 (2)	0.0136 (17)	−0.008 (2)
O3	0.066 (2)	0.067 (2)	0.0433 (18)	−0.0040 (17)	0.0108 (16)	−0.0020 (16)
I1	0.0854 (3)	0.0619 (2)	0.0487 (2)	0.00069 (17)	0.00906 (17)	0.00064 (14)
I2	0.0543 (2)	0.04402 (18)	0.05185 (19)	0.00195 (12)	0.00491 (13)	−0.00021 (12)
I3	0.0680 (2)	0.0673 (2)	0.0529 (2)	−0.00435 (15)	0.01033 (16)	−0.01723 (15)

### Geometric parameters (Å, °)

**Table:**

C1—C2	1.361 (6)	C6—H6	0.93
C1—C6	1.365 (6)	N1—O1	1.216 (5)
C1—N1	1.468 (5)	N1—O2	1.221 (5)
C2—C3	1.389 (5)	N2—H2A	0.89
C2—H2	0.93	N2—H2B	0.89
C3—C4	1.366 (6)	N2—H2C	0.89
C3—H3	0.93	O3—H3A	0.88 (2)
C4—C5	1.362 (6)	O3—H3B	0.89 (5)
C4—N2	1.480 (5)	I1—I2	2.8982 (6)
C5—C6	1.398 (6)	I2—I3	2.9694 (6)
C5—H5	0.93		
			
C2—C1—C6	123.6 (4)	C1—C6—C5	118.0 (4)
C2—C1—N1	118.3 (4)	C1—C6—H6	121
C6—C1—N1	118.1 (4)	C5—C6—H6	121
C1—C2—C3	118.4 (4)	O1—N1—O2	123.9 (4)
C1—C2—H2	120.8	O1—N1—C1	118.9 (4)
C3—C2—H2	120.8	O2—N1—C1	117.1 (4)
C4—C3—C2	118.4 (4)	C4—N2—H2A	109.5
C4—C3—H3	120.8	C4—N2—H2B	109.5
C2—C3—H3	120.8	H2A—N2—H2B	109.5
C5—C4—C3	123.3 (4)	C4—N2—H2C	109.5
C5—C4—N2	119.0 (4)	H2A—N2—H2C	109.5
C3—C4—N2	117.7 (4)	H2B—N2—H2C	109.5
C4—C5—C6	118.4 (4)	H3A—O3—H3B	114 (6)
C4—C5—H5	120.8	I1—I2—I3	178.209 (14)
C6—C5—H5	120.8		
			
C6—C1—C2—C3	0.7 (7)	C2—C1—C6—C5	−2.3 (7)
N1—C1—C2—C3	−179.3 (4)	N1—C1—C6—C5	177.7 (4)
C1—C2—C3—C4	1.2 (7)	C4—C5—C6—C1	2.0 (6)
C2—C3—C4—C5	−1.5 (7)	C2—C1—N1—O1	−164.1 (4)
C2—C3—C4—N2	176.4 (4)	C6—C1—N1—O1	15.9 (6)
C3—C4—C5—C6	−0.2 (7)	C2—C1—N1—O2	16.7 (6)
N2—C4—C5—C6	−178.0 (4)	C6—C1—N1—O2	−163.2 (4)

### Hydrogen-bond geometry (Å, °)

**Table:**

*D*—H···*A*	*D*—H	H···*A*	*D*···*A*	*D*—H···*A*
N2—H2A···O3^i^	0.89	1.94	2.824 (5)	173
N2—H2B···I3^ii^	0.89	3.01	3.731 (4)	139
N2—H2C···O1^iii^	0.89	2.52	2.922 (5)	108
N2—H2C···O3^iii^	0.89	2.02	2.860 (5)	157
O3—H3A···O2	0.88 (2)	1.98 (3)	2.818 (5)	158 (6)
O3—H3B···I1^iv^	0.89 (5)	2.88 (5)	3.722 (3)	157 (4)

Symmetry codes: (i) *x*−1, −*y*+1/2, *z*+1/2; (ii) −*x*, −*y*+1, −*z*+1; (iii) *x*, −*y*+1/2, *z*+1/2; (iv) −*x*+1, −*y*+1, −*z*.
